# Comparing survival rates and mortality in operative versus nonoperative treatment for femoral neck fractures among Alzheimer's disease patients: A retrospective cohort study

**DOI:** 10.1002/agm2.12279

**Published:** 2023-12-19

**Authors:** Yijiong Yang, Stacy A. Drake, Jing Wang, Gordon C. Shen, Hongyu Miao, Robert O. Morgan, Xianglin L. Du, David R. Lairson

**Affiliations:** ^1^ College of Nursing Florida State University Tallahassee Florida USA; ^2^ School of Nursing Bowling Green State University Bowling Green Ohio USA; ^3^ Department of Management, Policy and Community Health, School of Public Health The University of Texas Health Science Center at Houston Houston Texas USA; ^4^ Department of Statistics Florida State University Tallahassee Florida USA; ^5^ Department of Epidemiology, Human Genetics and Environmental Sciences, School of Public Health The University of Texas Health Science Center at Houston Houston Texas USA

**Keywords:** Alzheimer's disease and Alzheimer's disease related dementias, arthroplasty, femoral neck fracture, internal fixation, non‐operative treatment

## Abstract

**Introduction:**

Addressing femoral neck fractures resulting from ground‐level falls in older adults with Alzheimer's disease (AD) involves a personalized treatment plan. There is considerable ongoing debate concerning the relative advantages and disadvantages of surgical treatment (internal fixation or arthroplasty) vs nonoperative treatment for femoral neck fractures in older persons with AD.

**Methods:**

This retrospective cohort study compared the mortality, hazard ratio, and survival rate between operative and nonoperative treatments, controlling for patients' demographic information and baseline health status. The study population consisted of Optum beneficiaries diagnosed with AD who experienced an initial femoral neck fracture claim between January 1, 2012, and December 31, 2017. Kaplan–Meier survival curves were applied to compare the treatment groups' post‐fracture survival rates and mortality. Cox regression was used to examine the survival period by controlling the covariates.

**Results:**

Out of the 4157 patients with AD with femoral neck fractures, 59.8% were women (n = 2487). The median age was 81 years. The 1‐year survival rate for nonoperative treatment (70.19%) was lower than that for internal fixation (75.27%) and arthroplasty treatment (82.32%). Compared with the nonoperative group, arthroplasty surgical treatment had significant lower hazard risk of death (arthroplasty hazard ratio: 0.850, 95% CI: 0.728–0.991, *P* < 0.05).

**Discussion:**

The findings suggest that the operative treatment group experiences higher survival rates and lower mortality rates than the nonoperative group. This paper provides insights into treatment outcomes of older adults with AD receiving medical care for femoral neck fractures.

## Graphical abstract

Addressing femoral neck fractures resulting from ground‐level falls in older adults with Alzheimer's disease (AD) involves personalized treatment plan. This retrospective cohort study examined Optum beneficiaries diagnosed with AD who had femoral neck fracture claim. The findings suggested that operative treatment group experiences higher survival rates and lower mortality rates compared to the nonoperative group.

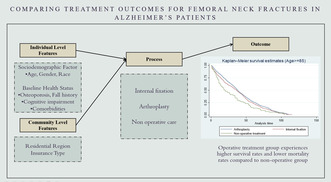

## 1 BACKGROUND

Alzheimer's disease (AD) is an incurable chronic neurodegenerative disease with a progressive pattern of cognitive impairment, behavioral dysfunction, and psychiatric symptoms.^1^ Approximately 5.5 million Americans are currently living with AD, including 5.3 million seniors over 65 years of age. By 2050, this number is estimated to reach nine million.^2^ As memory loss symptoms progress, the disease can be divided into four stages (cognitive impairment, mild, moderate, and severe). During the preclinical and early stages, a person may experience mild cognitive difficulties and memory loss.^3^ As the AD advances, people are progressively unable to perform common activities of daily living and have an increased risk of falling, which may result in injuries such as hip fractures.^4^ With late‐stage AD, individuals lose the ability to write, read, or respond to a conversation and cannot perform daily living tasks independently.^5^

In the United States, each year, more than 350,000 patients aged over 65 years visit emergency departments with hip fractures, constituting 86% of all reported hip fractures occurring in individuals.^6^, ^7^ More than 95% of hip fractures are caused by ground‐level falls.^8^ Individuals with AD have a higher incidence of falls resulting in injury and face a greater risk for delirium and a higher mortality rate after injury compared to individuals without AD.^9^, ^10^ The incidence of hip fracture among patients with and without AD was 17.4 and 6.6 per 1000 person‐years.^4^, ^11^ Research has demonstrated that individuals with AD who experience a hip fracture may lose their ability to move independently, resulting in an increased length of stay and higher medical costs in the hospital and long‐term care facilities.^4^, ^12^, ^13^, ^14^, ^15^ Moreover, individuals with AD following a hip fracture may require an extended period for rehabilitation and encounter challenges in ambulatory improvement due to cognitive difficulties, reducing the likelihood of recovering their previous functional status.^10^, ^16^, ^17^, ^18^ Several factors contribute to the association between AD and hip fracture, including the risk of ground‐level falls, weight loss, muscle weakness, and osteoporosis.^16^ Osteoporosis is a disease that causes bones to become weak. For older populations with osteoporosis aged between 60 and 85 years, the risk of fracture doubles for every 5‐ to 6‐year increase in age.^7^

Surgical repair, utilizing internal fixation (insertion of several screws to stabilize the broken hip) or arthroplasty (replacement of part or the entire hip/femur joint), is the standard for most patients with femoral neck hip fractures. However, a small proportion of patients who are medically unfit for surgeries may undergo nonoperative treatment, involving bed rest followed by physical rehabilitation and medications to prevent blood clots and release pain. This conservative approach is adopted when the risks of surgery outweigh the benefits.^19^, ^20^

There is considerable ongoing debate concerning the merits and drawbacks of surgical repairs compared to nonoperative approaches for femoral neck fractures in patients with AD.^21^, ^22^ According to van de Ree's systematic review/meta‐analysis on hip fractures in people with AD, only a few studies with a small number of patients comparing surgery with nonoperative treatments have been published.^23^ Nonoperative treatment had a higher risk of mortality,^22^ but could be a viable choice for patients with limited life expectancy and who lack the ability to gain improved postoperational functional status.^24^, ^25^, ^26^ The nonoperative treatment group did not demonstrate inferiority to operative management regarding health related quality of life.^25^ Arthroplasty caused a decline in postoperative capacity mobility and balance capacity, especially in patients with mental dysfunction or severe comorbidities.^27^

This paper aims to compare and assess the post fracture mortality, survival rates, and hazard ratios of patients with AD with femoral neck fractures undergoing operative surgery (internal fixation/arthroplasty surgery) or nonoperative conservative treatment.

## 2 METHODS

This retrospective cohort study compared the survival rates, mortality rates, and hazard ratios between operative treatment and nonoperative treatment, controlling for the patient's demographic information and baseline health status covariates (age, gender, race, pre‐fracture residence region, baseline health status covariates, and insurance types). This study included Optum beneficiaries with an AD diagnosis who had a claim for femoral neck fracture between January 1, 2012, and December 31, 2017. Patients with multiple hip fractures between January 1, 2011, and December 31, 2011, were excluded to ensure that only patients with first time hip fractures were included. Patients diagnosed with AD were identified using International Classification of Disease 9th and 10th revisions (ICD‐9/10) diagnosis codes before the diagnosis of a femoral neck fracture. Patients were categorized according to types of treatment: internal fixation, arthroplasty surgery, and nonoperative treatment. Claims records with diagnosis and procedure codes corresponding to femoral neck fracture were included; a combination of hip fracture sections and pathological hip fractures were excluded. The combination of intracapsular and extracapsular fracture is rare and without optimal surgical solution.^28^

### 2.1 Sampling population

Beneficiaries diagnosed with AD who experienced their first femoral neck fracture between January 1, 2012, and December 31, 2017, were identified through claims data.^29^

Inclusion criteria: (1) Patients with AD (ICD‐9 codes 331.0 and ICD‐10 G30.0–G30.9); (2) patients with AD with a claim for initial femoral neck fracture between January 1, 2012, and December 31, 2017; (3) patients treated with nonoperative management for femoral neck fracture; (4) patients treated with internal fixation surgery for femoral neck fracture; (5) patients with arthroplasty surgery for femoral neck fracture, and (6) patients have continuous health plan coverage for at least 1 year before and 1 year after femoral neck fracture or death (whichever occurred earlier).

Exclusion criteria: (1) Patients with AD with multiple femoral neck fracture history (any ICD–9/10 diagnosis codes corresponding to femoral neck fracture) between January 1, 2011, and December 31, 2011, to avoid re‐admitted cases; (2) individuals without continuous coverage for at least 6 months after diagnosis of femoral neck fracture; (3) patients with AD with pathological hip fractures as specified by ICD‐9/10 coding, and (4) patients with AD with a combination of hip fractures (eg, a combined intracapsular and extracapsular fracture).

Baseline health status indicators identified between January 1, 2012, and December 31, 2017, served as covariates. According to the literature review, comorbidities related to AD and femoral neck fracture include^9^: a history of falls, osteoporosis, depression, abnormal weight loss, sarcopenia/cachexia/wasting atrophy/muscle weakness, malaise and fatigue, and depression. Charlson Comorbidity Index (CCI) was included in the Cox regression survival analysis. The CCI is a method for categorizing comorbidities based on all the ICD–9–CM and ICD–10–CM diagnosis codes between January 1, 2012, and December 31, 2017.^30^ Several comorbidity categories were included in the CCI: congestive heart failure, myocardial infarction, peripheral vascular disease, cerebrovascular disease, dementia (chronic cognitive deficit or a low level of dementia), chronic pulmonary disease, connective tissue disease‐rheumatic disease, peptic ulcer disease, diabetes without and with chronic complications, paraplegia and hemiplegia, renal disease, cancer, mild, moderate or severe liver disease, metastatic carcinoma, and HIV/AIDS. A lower CCI score indicated mild comorbid conditions and a higher score indicated severe comorbid conditions. This research categorized the CCI into three grades: mild with CCI scores of 1–2; moderate with CCI scores of 3–4; and severe with CCI scores ≥ 5.

The length of survival was computed from hospice claims, eligibility end date, and last claim date. Patients with at least one hospice claim were presumed to have died; the date of the previous medical claim was used as a proxy for the date of death. Survival time after diagnosis was defined as the time between the operative or nonoperative treatment date and the proxy date of death.

Facility and professional claims included patient demographic and health care service information, such as patients' age, gender, race, residence region, insurance type, and baseline health status. The Chi‐squared test and Kruskal‐Wallis test were used to compare the differences for covariates between different treatment groups, with a significance level of 0.05 as the threshold of balance between groups. Kaplan–Meier method functions were applied to compare the post‐fracture survival rates between the treatment groups. Cox regression was applied to examine the survival period by controlling the covariates. Excel and Stata 15.0 were used for data management, storage, cleaning the dataset, and data analysis. The significance level was set at 0.05. The UTHealth Committee for the Protection of Human Subjects approved the study.

## 3 RESULTS

A total of 4157 patients with AD with femoral neck fractures were selected from claims data, including 1508 patients who underwent internal fixation, 2334 arthroplasty surgery cases, and 315 nonoperative treatment cases; nearly 60% of them were women (n = 2487, 59.8%), and 1668 (40.1%) of them were men. The median age was 81 (interquartile range: 75–87). The frequency of patients with AD with femoral neck fracture was highest among White individuals (n = 2726, 65.6%), followed by 734 (17.7%) African American individuals, 443 (10.7%) Hispanics, and 76 (1.8%) Asians. Over 90% of individuals were covered by Medicare. Over 40% (n = 1722) of the individuals lived in the south region, 960 (23.1%) lived in the mid‐west region, 866 (20.8%) lived in the northeast region, and 606 (14.6%) lived in the west region. Over half of the individuals had a pre‐existing history of atrophy/muscle weakness, malaise, and fatigue. Around half of arthroplasty cases (n = 174, 55.2%) had muscle weakness and fatigue. However, individuals in the nonoperative group had significantly less history of falls, osteoporosis, atrophy/muscle weakness, malaise and fatigue, abnormal weight loss, and depression compared to the surgery treatment group. The health condition of the operative group was worse than the non‐operative group (Table 1).

**TABLE 1:** Characteristics of patients with Alzheimer's disease with femoral neck fractures

	Total (n = 4157)	Internal fixation (n = 1508)	Arthroplasty (n = 2334)	Nonoperative treatment (n = 315)	*P* value
Total					
Age,^a^ median (IQR) in y	81 (75–87)	82 (75–87)	81 (75–87)	80 (73–86)	0.020*
Sex,^b^ No. (%)					
Male	1668 (40.1)	588 (39.0)	960 (41.1)	120 (38.1)	0.300
Female	2487 (59.8)	920 (61.0)	1372 (58.8)	195 (61.9)	
Unknown	2 (0.1)	0 (0.0)	2 (0.1)	0(0.0)	
Race,^b^ No. (%)					
White	2726 (65.6)	982 (65.1)	1540 (66.0)	204 (64.8)	0.050
Black	734 (17.7)	248 (16.4)	438 (18.8)	48 (15.2)	
Hispanic	443 (10.7)	180 (11.9)	222 (9.5)	41 (13.0)	
Asian	76 (1.8)	26 (1.7)	41 (1.8)	9 (2.9)	
Unknown	178 (4.3)	72 (4.8)	93 (4.0)	13 (4.1)	
Residential state^b^ (US census region), No. (%)					
West	606 (14.6)	254 (16.8)	288 (12.3)	64 (20.3)	< 0.001**
Midwest	960 (23.1)	302 (20.0)	603 (25.8)	55 (17.5)	
South	1722 (41.4)	638 (42.3)	944 (40.5)	140 (44.5)	
Northeast	866 (20.8)	314 (20.8)	498 (21.3)	54 (17.1)	
Unknown	3 (0.1)	0 (0.0)	1 (0.1)	2 (0.6)	
Insurance type,^b^ No. (%)					
Medicare	3886 (93.5)	1416 (93.9)	2189 (93.8)	281 (89.2)	0.010*
Commercial	271 (6.5)	92 (6.1)	145 (6.2)	34 (10.8)	
History of fall,^b^ No. (%)	1800 (43.3)	573 (38.0)	1167 (50.0)	60 (19.0)	< 0.001**
Osteoporosis,^b^ No. (%)	1092 (26.3)	412 (27.3)	636 (27.2)	44 (14.0)	< 0.001**
Sarcopenia/cachexia/wasting atrophy/muscle weakness,^b^ No. (%)	2655 (63.9)	843 (55.9)	1699 (72.8)	113 (35.9)	< 0.001**
Malaise and fatigue,^b^ No. (%)	3348 (80.5)	1181 (78.3)	1993 (85.4)	174 (55.2)	< 0.001**
Abnormal weight loss,^b^ No. (%)	1112 (26.8)	425 (28.2)	642 (27.5)	45 (14.3)	< 0.001**
Depression,^b^ No. (%)	2034 (48.9)	661 (43.8)	1271 (54.5)	102 (32.4)	< 0.001**

Abbreviation: IQR, interquartile range.

^a^ Kruskal‐Wallis test was used to determine statistically significant difference of age between operative surgery (internal fixation, arthroplasty) or non‐operative conservative treatment groups.

^b^ Chi‐Squared test was performed to check significance of gender, race, residential region, insurance plan, history of fall, and osteoporosis between operative surgery (internal fixation, arthroplasty) or non‐operative conservative treatment groups.

* *P* value less than 0.05.

** *P* value less than 0.001.

**FIGURE 1:**
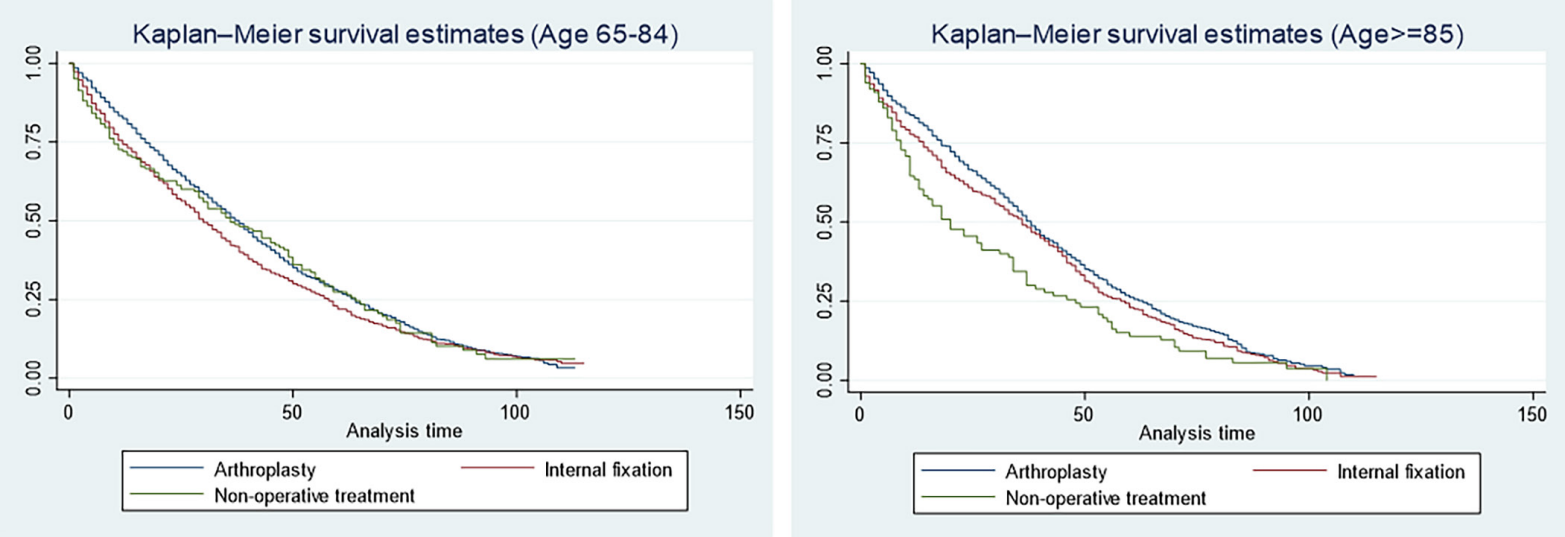
Survival curves for femoral neck fractures among patients with Alzheimer's disease (in months).

Figure 1 displays the Kaplan–Meier survival curves for different age groups. Among individuals aged 65–84, the 1‐year survival rate was higher in the arthroplasty surgery group than in the internal fixation and nonoperative treatment groups. Significant disparities in survival curves were evident for the age group over 85 years, with the nonoperative treatment group exhibiting a higher risk of death in contrast to the surgical group (Figure 1). In the nonoperative treatment group, the 1‐year survival rate was 70.19%, lower than the internal fixation group (75.27%) and the arthroplasty treatment group (82.32%). Notably, nonoperative treatment exhibited a considerable overall mortality of 30% within the initial 12 months. The median survival time for arthroplasty cases reached approximately 3 years, whereas the nonoperative group demonstrated a median survival time of approximately 2.5 years (Figure 1).

Table 2 presents each treatment group's cumulative time at risk, mortality rates, and individual median survival times. The arthroplasty surgical group exhibited the lowest mortality rate throughout the entire study period compared to the internal fixation and nonoperative groups (Mortality rates per 100,000 person months: 2150 vs. 2430 vs. 2460). The median survival time for the overall 4157 included patients with AD was 36.5 months. Notably, arthroplasty surgery yielded the best median survival time compared to internal fixation and nonoperative treatments. Specifically, the median survival time for the arthroplasty treatment group was 38 months, surpassing the median survival time of 33 months for individuals who underwent internal fixation or nonoperative treatment following the fracture.

**TABLE 2:** Mortality rates for femoral neck fractures among patients with Alzheimer's disease

	Time at risk^a^ (person month)	Mortality rate^b^ (per 100,000 person months)	Survival time in months		
			25th Percentile	50th Percentile	75th Percentile
Nonoperative treatment	9402	2460	10	33	59
Internal fixation	50,464	2430	13	33	59
Arthroplasty	88,782	2150	18	38	64

^a^ Total amount of time during which participants are considered at‐risk and under observation for the outcome of interest. Unit: person month.

^b^ Mortality rate of femoral neck fracture during the observation period. Unit: per 100,000 person months.

The multivariable Cox proportional hazards model was applied to estimate the effects of factors on survival time. The covariate factors evaluated in this model included age, gender, race, residential region, insurance type, and the CCI. Cox regression with CCI scores adjustment, hazard ratio (HR) and confidence interval (CI) level of 95% were summarized in Table 3. Compared with the nonoperative group, internal fixation, and arthroplasty surgical treatment had lower hazard death risk (internal fixation HR: 0.961, 95% CI: 0.821–1.125, *P* = 0.624; arthroplasty HR: 0.850, 95% CI: 0.728–0.991, *P* < 0.05). The HRs that exhibited significance include age at 1.006 (95% CI: 1.001–1.009, *P* < 0.05), male gender at 1.233 (95% CI: 1.149–1.324, *P* < 0.001), and Medicare insurance coverage at 1.200 (95% CI: 1.036–1.391, *P* < 0.05). Higher CCI scores (≥ 5) showed higher HRs (HR: 1.039, 95% CI: 0.848–1.272, *P* = 0.713) in contrast to lower CCI scores (refer to Table 3).

**TABLE 3:** Adjusted Cox regression survival rates of femoral neck fracture among patients with Alzheimer's disease

	Hazard ratio	*Z*	95% Confidence interval	*P* value
Age (y)	1.006	2.55	1.001–1.009	0.011*
Sex				
Female (Reference group)	1.000			
Male	1.233	5.78	1.149–1.324	< 0.001**
Race				
White (Reference group)	1.000			
Black	0.941	−1.28	0.857–1.033	0.202
Hispanic	0.995	−0.09	0.892–1.110	0.930
Asian	0.915	−0.58	0.678–1.235	0.561
Residential state (US census region)				
Northeast (Reference group)	1.000			
South	1.042	0.87	0.949–1.144	0.387
Midwest	1.009	0.17	0.911–1.118	0.862
West	0.868	−2.26	0.768–0.981	0.024*
Insurance type				
Commercial (Reference Group)	1.000			
Medicare	1.200	2.44	1.036–1.391	0.015*
Charlson Comorbidity Index				
1–2 (Reference group)	1.000			
3–4	0.954	−0.42	0.766–1.190	0.678
≥5	1.039	0.37	0.848–1.272	0.713
Treatment type				
Nonoperative (Reference group)	1.000			
Internal fixation	0.961	−0.49	0.821–1.125	0.624
Arthroplasty	0.850	−2.07	0.728–0.991	0.038*

* *P* value less than 0.05.

** *P* value less than 0.001.

## 4 DISCUSSION

Many studies have demonstrated that surgical treatment compared to nonoperative surgical treatment provides better outcomes in treating femoral neck fractures.^22^ A recent study performed in one local hospital in Singapore showed a high risk of dying after a hip fracture in nonoperative management; the 1‐year mortality rate of nonoperative treatment was four times higher than the operative group.^22^ This finding is consistent with the difference in survival rate in this study. This study showed that the 1‐year survival rate of nonoperative treatment (70.19%) was lower than the internal fixation (75.27%) and arthroplasty treatment (82.32%). Nonoperative treatment showed an overall mortality of 30% among patients with AD with fractures within the first 12 months. Arthroplasty treatment had the lowest 1‐year mortality rate and longer median survival time compared with internal fixation and nonoperative treatment. After controlling patients' age, gender, race, insurance type, CCI, and residential region, the arthroplasty surgery treatment showed significant lower HRs than the nonoperative treatment (arthroplasty HR: 0.850, 95% CI: 0.728–0.991, *P* < 0.05). Other researchers^31^ studied 758 patients with femur fracture treated in one level 3 community hospital and found that the overall unadjusted 1‐year mortality rate was 21.2%. Our overall 1‐year morality rate was 21.1%, which aligns with the previous study.

The incidence rate of femoral neck fracture increases with age and life expectancy.^32^ Hip fractures could heal without surgery, but patients without surgery could lay in bed for several weeks or months.^23^ However, prolonged bed rest has a greater risk of complications. Operative surgery allows patients to leave the bed within a shorter period after surgery. Nevertheless, some patients may refuse to undergo surgery because of different reasons (economic burden, functional loss, and pain after surgery).^33^ Surgery is the most frequently refused intervention, and the most common reason for refusal is side effects after surgery.^34^ These reasons are, in part, why conservative nonoperative treatment is an alternative.

Although operative management plays the leading role in treating patients with hip fractures, patients with multiple comorbid conditions are sometimes treated nonoperatively to avoid worsening their status due to their comorbidities.^34^ Nonoperative treatment could be a better choice for patients who cannot gain improved postoperational functional status compared with pre‐surgery.^24^ In this paper, nonoperative treatment for patients with hip fractures was associated with increased mortality compared to the operative treatment group. This finding was in line with other articles. We also found that patients in the nonoperative treatment group had fewer comorbidities, and their baseline health conditions were better than patients who underwent internal fixation and arthroplasty. Only 14% of the nonoperative cases had osteoporosis compared with 27% of the internal fixation and arthroplasty cases had osteoporosis. A potential reason for this is that patients with better health conditions can meet the requirement for nonoperative care and could have better post‐treatment outcomes compared with patients with AD with multiple comorbid conditions; however, operative management was still the most effective treatment.

Dementia is strongly associated with osteoporosis and osteoporotic fracture.^35^ The presence of dementia increases hip fracture incidence via intermediate risk factors, such as falls and osteoporosis.^9^ Ha et al^36^ demonstrated the factors that affected the 1‐year mortality in patients with hip fracture were age [odds ratio (OR): 1.06, *P* < 0.001], sex (OR: 2.68, *P* < 0.001), CCI (OR: 1.34, *P* < 0.001), and dementia (OR: 1.70, *P* = 0.016). In this paper, we had similar findings. Age is an important risk factor for mortality following a fracture. Patients with AD aged over 85 years who had a femoral neck fracture were at a significantly greater risk of dying after electing nonoperative treatment compared to either surgical treatment group.

A limitation of this study is that the cause of mortality is not defined. For example, a patient could have a femoral neck fracture but die because of heart disease, wound infections, or other comorbidities. Another limitation is potential selection bias due to the unobservable differences and factors between treatment types, which may influence the survival rates of patients after fracture. There may be some misdiagnoses, given the difficulty of differentiating AD from other forms of dementia. Dementia most commonly results from AD (60%–89%).^30^ Dementia's clinical presentations differ based on etiology, and hip fracture incidence differs based on etiology.^9^ In this study, all patients with AD were categorized within one group without attention to disease progression at the time of fracture. However, several papers found that the risk of femur neck fracture remained constant in mild, moderate, and severe AD. Dementia severity was not significantly predictive of fractures.^9^

The cause of mortality will be included in the future study. The provider facility type, provider service location, and the number of hospital beds are associated with treatments (surgery vs non‐surgery) and total medical costs.^37^ For example, general hospitals in large metropolitan areas may have different treatment patterns and costs than hospitals in non‐metropolitan areas. Future research will assess the effect of provider factors on the treatment patterns, medical care cost, and mortality rates for femoral neck fractures among patients with AD.

## 5 CONCLUSION

The survival rate of patients undergoing operative treatment following a hip fracture was notably higher than that of the nonoperative group, particularly among patients aged over 85 years. Arthroplasty treatment exhibited the lowest incidence rate and a longer median survival time compared to internal fixation and nonoperative treatment. Although patients in better health may qualify for nonoperative approaches and potentially achieve better post‐treatment outcomes, this study underscores the effectiveness of operative management. Focusing on femoral neck fractures in individuals with AD, this paper recognizes the complexity of these patients' medical comorbidities, mental conditions, functional limitations, and increased mortality and health care burden. The analysis of all‐cause mortality, survival rates, and HRs related to operative and nonoperative treatments for femoral neck fractures in patients with AD offers valuable insights for health care providers, researchers, policymakers, and managers. By enhancing treatment assessment and efficiency, this study contributes to aging, clinical care, and geriatrics research.

## AUTHOR CONTRIBUTIONS

Y.Y. and D.L. were responsible for the protocol design of the research. Y.Y. extracted and analyzed the data. Y.Y. wrote the first draft of the manuscript. S.D., J.W., G.S., H.M., R.M., and X.D. helped with the revisions of the article. All authors reviewed the study, read, and approved the final manuscript.

## FUNDING INFORMATION

There is no funding to report.

## CONFLICT OF INTEREST STATEMENT

The authors declare that they have no competing interests.

## ETHICS STATEMENT

Ethical approval for the study was obtained from the UTHealth Committee for the Protection of Human Subjects (HSC‐SPH‐21‐0690). This secondary research utilized de‐identified pre‐existing data. Waiver of consent was granted, and the study was exempted from the Health Insurance Portability and Accountability Act.

## Data Availability

The data that support the findings of the current study were available from the UTHealth School of Public Health Center for Health Care Data. Still, restrictions may apply to the availability of these data, which were under the permission of UTHealth School of Public Health.
